# Application of Pender’s health promotion model for type 2 diabetes treatment adherence: protocol for a mixed methods study in southern Iran

**DOI:** 10.1186/s13063-022-07027-9

**Published:** 2022-12-28

**Authors:** Nahid Shahabi, Zahra Hosseini, Teamur Aghamolaei, Amin Ghanbarnejad, Ahmad Behzad

**Affiliations:** 1 Student Research Committee, Hormozgan University of Medical Sciences, Bandar Abbas, Iran; 2 Social Determinants in Health Promotion Research Center, Hormozgan Health Institute, Hormozgan University of Medical Sciences, Bandar Abbas, Iran; 3 Cardiovascular Research Center, Hormozgan University of Medical Sciences, Bandar Abbas, Iran

**Keywords:** Type 2 diabetes mellitus, Treatment adherence, Mixed method study

## Abstract

**Background:**

Type 2 diabetes (T2D) mellitus treatment as a chronic disease requires adequate adherence to treatment including controlling blood glucose levels and lifestyle management. The aim of this study is to investigate the factors affecting of adherence to T2D treatment from the perspective of patients and design an intervention program based on Pender’s health promotion model (HPM) to increase T2D treatment adherence in Bandar Abbas, a city located in the south of Iran.

**Methods:**

This mixed method study will consist of qualitative stage, questionnaire design and a randomized, open-label, parallel-group interventional study based on HPM in southern Iran. Sampling for qualitative stage will continue until reaching the saturation. In the intervention stage, participants will be 166 T2D patients referring to the Bandar Abbas Diabetes Clinic will be randomized into intervention and control groups (allocation 1:1). After identifying the factors affecting adherence to treatment in T2D patients by qualitative study and literature review, a questionnaire based on HPM will be designed. In the next stage, 10 sessions of intervention for the intervention group will be designed. To evaluate the effect of the intervention, intervention and control groups will be tested for hemoglobin A1c (HbA1c) before and 3 months after the intervention.

**Discussion:**

This designed study is a program for improving treatment adherence in T2D based on the HPM model and contributes to a better understanding of effective factors in adherence to treatment in T2D patients. The results of this project can be used for macro-diabetic planning.

**Trial registration:**

This study is registered on the Iranian Registry of Clinical Trials (IRCT20211228053558N1: https://www.irct.ir/trial/61741) and first release date of 17th March 2022.

**Supplementary Information:**

The online version contains supplementary material available at 10.1186/s13063-022-07027-9.

## Administrative information

Note: the numbers in curly brackets in this protocol refer to SPIRIT checklist item numbers. The order of the items has been modified to group similar items (see http://www.equator-network.org/reporting-guidelines/spirit-2013-statement-defining-standard-protocol-items-for-clinical-trials/).

**Table:** 

Title {1}	Application of Pender’s health promotion model for Type 2 diabetes treatment Adherence: Protocol for a Mixed Methods Study in southern Iran
Trial registration {2a and 2b}.	IRCT (IRCT20211228053558N1: https://www.irct.ir/trial/61741) Registered on 17th March 2022.
Protocol version {3}	Version 1 of 17th March 2022
Funding {4}	This study is supported by a research grant from the Hormozgan University of Medical Sciences. The funding body (HUMS) didn’t have any role in the design of the study and collection, analysis, and interpretation of data and in writing the manuscript.
Author details {5a}	Nahid Shahabi: Student Research Committee, Faculty of Health, Hormozgan University of Medical Sciences, Bandar Abbas, Iran. Zahra Hosseini: Social Determinants in Health Promotion Research Center, Hormozgan Health Institute, Hormozgan University of Medical Sciences, Bandar Abbas, Iran. Teamur Aghamolaei: Cardiovascular Research Center, Hormozgan University of Medical Sciences, Bandar Abbas, Iran. Amin Ghanbarnejad: Social Determinants in Health Promotion Research Center, Hormozgan Health Institute, Hormozgan University of Medical Sciences, Bandar Abbas, Iran. Ahmad Behzad: Social Determinants in Health Promotion Research Center, Hormozgan Health Institute, Hormozgan University of Medical Sciences, Bandar Abbas, Iran.
Name and contact information for the trial sponsor {5b}	Hormozgan University of Medical Sciences: info@hums.ac.ir
Role of sponsor {5c}	The sponsors played no role in the design of the study and will not play any role in the collection, management, analysis, or interpretation of the data. They do not have ultimate authority over any of these activities.

## Introduction

### Background and rationale {6a}

Type 2 diabetes mellitus (T2D), one of the most common global chronic diseases, is a serious threat to the public health and healthcare systems [1]. According to the 10th edition of the IDF Diabetes Atlas, it was estimated that there are 536.6 million people with diabetes worldwide in 2021 and were expected to increase to 783.2 million by 2045 [2]. The prevalence of diabetes mellitus in Iran is 11.4% among 25–70 years [3], and expectation until 2030 is 9.24 million cases (6.73 million diagnosed and 2.50 million undiagnosed) [4].

Treatment of T2D as a chronic disease requires adequate adherence to treatment: controlling blood glucose levels, and lifestyle management, including medical nutrition therapy, physical activity, and psychosocial care [5]. Adherence is defined as “the extent to which a person’s behavior, taking medication, following a diet, and/or executing lifestyle changes, corresponds with agreed recommendations from a health care provider” [6].

A systematic review study reported that in several developed countries treatment adherence in diabetic patients varies from 38.5 to 93.1% [7]. Control of hyperglycemia in Iran, despite the availability of medications and insulin coverage, is not sufficient [8]. A cross-sectional study (2021) reported 52.75% adherence to T2D treatment in Iran [9]. Treatment non-adherence in diabetic patients is associated with subsequent complications, increased mortality, increased treatment costs, reduced quality of life and even increased economic burden [7].

Since adherence to treatment in T2D is a complex behavior [10], theoretical-based qualitative studies can help researchers to understand the reason for this complex behavior and design suitable interventions [11]. Previous studies showed that theory-based health-promotion interventions can play an effective role in promoting healthy behavior in the diabetic patients [12, 13]. One of these models is Pender’s health promotion model (HPM). This model focuses on the role of experience in shaping behavior for explaining healthy behavior. HPM enables health professionals to discover a process that encourages people to adopt health-promotion behaviors, such as treatment adherence [14]. It includes three factors, namely, individual characteristics and experiences (including personal factors and previous behaviors), behavior-specific cognitions and affect (including structures such as perceived benefits and barriers and perceived self-efficacy), and behavioral outcomes (health-promoting behavior is the desired behavioral outcome and is the endpoint in the health promotion model). Behavior-specific cognition and affect includes the perceived benefits of behavior, perceived barriers to behavior, perceived self-efficacy, activity-related affect, and interpersonal and situational influences. They can lead to adherence to health-promotion behaviors compared to immediate competing demands and preferences [15]. Therefore, investigating these constructs in order to evaluate their impact on T2D patients’ treatment adherence can be useful in guiding us towards an effective intervention for these patients.

Considering the increasing prevalence of diabetes in Iran [16], and evidence gap in T2D in different areas of Iran, there is a need for more research, especially in remote regions [17]. Unhealthy lifestyle including physical inactivity and unhealthy diet is common in southern Iran [18], and this can be effective in T2D patients’ treatment adherence in this region. Therefore, the aim of this study is to investigate the factors affecting of adherence to T2D treatment (medication, diet and physical activity) from the perspective of patients and design an intervention program based on HPM to increase T2D treatment adherence in Bandar Abbas, a city located in the south of Iran.

### Objectives {7}

The objective of this study will be to

1. Understand the factors affecting to T2D patients’ treatment adherence at different levels from the perspective of patients
2. Design a valid and reliable instrument to measure T2D patients’ treatment adherence based on HPM
3. Design, implement, and evaluate intervention program to improve T2D patients’ treatment adherence based on HPM

### Trial design {8}

This mixed-method study consists of 3 phases.

Need assessment as the first step aims to identify the factors affecting adherence to treatment in T2D patients. Different qualitative and quantitative data sources will be used for need assessment. After combining the results of the need assessment, a questionnaire based on HPM to measure adherence to medication, physical activity, and diet will be designed. In the next stage, based on earlier phases the intervention will be designed. The intervention phase with a superiority design is a randomized, open-label, parallel-group study. The participants will be randomly assigned at a 1:1 ratio to control and intervention groups. The intervention group will participate in an intervention program to improve T2D treatment adherence treatment (medication, diet, and physical activity) based on the Pender health promotion model. To evaluate the effect of the intervention, intervention and control groups will be tested for hemoglobin A1c (HbA1c) before and after the intervention.

## Methods: participants, interventions, and outcomes

### Study setting {9}

**Fig. 1:**
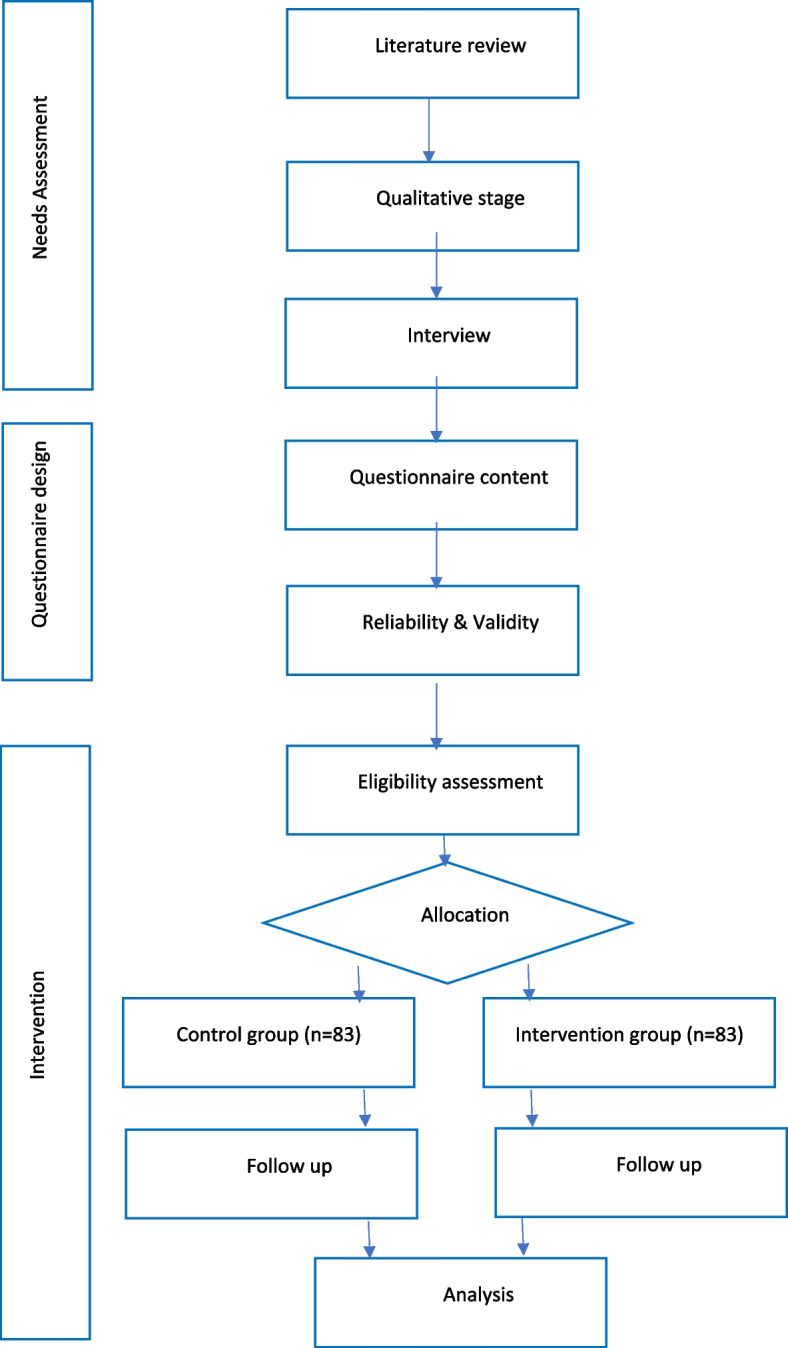
The study framework

To design this study, a research team consisting of three health education and promotion specialists, one doctor of internal medicine, and one biostatistics expert, is formed. The study population includes T2D patients referring to the Diabetes Clinic of Shahid Mohammadi Hospital in Bandar Abbas, and the place of study is the diabetes clinic of Shahid Mohammadi Hospital, the largest general hospital in Hormozgan province in the south of Iran. This clinic is the largest diabetes clinic in the province, where 1500 T2D patients have been registered. The study framework is summarized in Fig. 1.

### Eligibility criteria {10}

#### Qualitative stage

Eligible participants will be those who identify as women or men and are over the age of 80, have to be confirmed T2D, having rich and useful experiences of living with diabetes and their willingness to share their experiences with the researcher, and being able to participate in interviews and transfer information and experiences.

#### Intervention stage

Eligible participants will be women or men, over the age of 80, need to express their consent to participate in the research and have registered a medical case in the Diabetes Clinic of Shahid Mohammadi Hospital as a patient with T2D and also live in Bandar Abbas.

The exclusion criteria also include participating in intervention sessions (absence of more than two interventional sessions) and not referring to the Diabetes Clinic of Shahid Mohammadi Hospital.

#### Who will take informed consent? {26a}

The first author will enroll participants, will explain the purpose and process of intervention, and will obtain informed consent in both qualitative and intervention stages.

#### Additional consent provisions for collection and use of participant data and biological specimens {26b}

Participants will be requested to complete the consent form which includes items regarding consent to complete the questionnaire and consent to participate in the intervention: There are no charges from participants; they may withdraw or take away their permission to use the information at any time by sending a written notice to the researcher. If they withdraw their permission, they will exit the study. If necessary, any changes in the protocol modifications will be made with the consent of all authors and then, if finalized, will communicate to participants. There will be HbA1C test before and after the intervention.

### Interventions

#### Explanation for the choice of comparators {6b}

The designed intervention will be carried out only by the intervention group and the control group will only receive routine clinical care. The control group will receive training at the end of the study.

#### Intervention description {11a}

The intervention will be designed based on HPM constructs for T2D patients. The emphasis of the intervention materials will be on predictors of behavior that have been identified based on the need assessment and a pre-test that will be performed using the researcher-made questionnaire. The first author will recruit participants, will explain the purpose and process of intervention, and will obtain their informed consent to participate. According to the intervention program, the participants will not receive any other relevant education or training during the intervention. Sessions will present by one health education and promotion specialist and one internal specialist medicine, experienced in T2D treatment adherence. Intervention including 10 educational sessions in groups of 15 to 20 patients will be a combination of online and face-to-face methods according to the intervention group conditions. The intervention will be using challenging lectures, educational video clips, posters and pamphlets, and the main content of the sessions will be to promote and improve adherence to treatment in the areas of medication, diet, and physical activity. In-person sessions will be set to an average of 45 min and online sessions to 15 min. The control group will receive training at the end of the study.

#### Criteria for discontinuing or modifying allocated interventions {11b}

Leaving the study at any time by the participants will not affect the quality of services received in the diabetic clinic. Study participant collected data will be included in the analysis.

#### Strategies to improve adherence to interventions {11c}

The researchers will send intervention session reminders to the participants via short message service (SMS) and will be in touch with them. In case of missing sessions, the first author will contact the participant by phone call.

#### Relevant concomitant care permitted or prohibited during the trial {11d}

Relevant intervention or training during the intervention is prohibited. Usual routine clinical care health care is permitted during the study.

#### Provisions for post-trial care {30}

This is not applicable as there is no anticipated harm and compensation for trial participation.

#### Outcomes {12}

The primary outcome is treatment adherence which will be measured according to score of the researcher-made questionnaire and HbA1c test before and 3 months after the intervention. Secondary outcomes include attendance at clinic appointments, weight, healthy diet days, and physically active days.

#### Participant timeline {13}

Additional file is attached.

### Sample size {14}

#### Qualitative stage

Considering the fact that qualitative studies seek rich data and the greatest diversity, participants will not select solely from those who refer to the Diabetes Clinic of Shahid Mohammadi Hospital in Bandar Abbas (Bandar Abbas Diabetes Center). Sampling will be carried out using a maximum variation of the purposive sample (in terms of age, educational status, marital status, employment status, diabetes status, and adherence to treatment status). Sampling will continue without any limit on the number of samples until reaching the saturation stage. We will use the information from the medical files registered with the Diabetes Clinic of Shahid Mohammadi Hospital in Bandar Abbas to identify eligible patients

#### Intervention stage

When designing the educational intervention, the two intervention and control groups will be selected from T2D patients with registered medical cases in the Diabetes Clinic of Shahid Mohammadi Hospital in Bandar Abbas. According to previous study [19], the pooled standard deviation is calculated to be 29.7. Also, assuming a type I error = 5%, test power = 80%, and also considering a 13.5-score difference in adherence to treatment of the intervention and control group, the sample size of each group is estimated to be equal to 76 [20]. Considering the 10% possible drop-out, the sample size is determined 83 people in each of the two groups.

$$n=\frac{2{\left({z}_{1-\frac{a}{2}}^2+{z}_{1-\beta}\right)}^2{s}_p^2}{{\left({\mu}_1-{\mu}_2\right)}^2}=76$$

#### Recruitment {15}

The first author arranges appointments to explain the study process to the patients and requests them to complete the informed consent form if they are eligible. Needs assessment as the first stage started in February 2022, and the expected recruitment of patients referred to Diabetes Clinic of Shahid Mohammadi Hospital in Bandar Abbas for intervention will be in November 2022. The study is anticipated to continue until March 2023.

### Assignment of interventions: allocation

#### Sequence generation {16a}

Randomization will be done through systematic random sampling by a biostatistics expert according to the list of participants (whose eligibility has been confirmed and registered in the diabetes clinic). Then, selected participants will randomly be assigned in a 1:1 ratio to the control group or intervention group.

#### Concealment mechanism {16b}

Details of the allocation process will be concealed. The randomizer and first and second authors are informed of the randomization result.

#### Implementation {16c}

After obtaining signed informed consent forms, the first author will generate the allocation sequence, enroll, and assign participants to interventions.

### Assignment of interventions: blinding

#### Who will be blinded {17a}

The researchers, patients, and biostatistics expert will not be blinded.

#### Procedure for unblinding if needed {17b}

Since this study is designed open-labeled, there is no blinding method.

## Data collection and management

### Plans for assessment and collection of outcomes {18a}

#### Literature review

Databases such as Web of Science, PubMed, Elsevier, Scopus, and Medline, as well as the Google Scholar, will be explored for related studies on the factors affecting adherence to treatment in T2D patients.

#### Qualitative stage

In order to collect data for identifying factors affecting adherence to treatment in T2D patients, in-depth individual and semi-structured interviews with patients in Bandar Abbas will be used. In this step, the directed content analysis method will be used. After obtaining ethical approval and informed written consent from all participants, interviews will be conduct by first author. An interview guide (Table 1) is developed for the interviews, which includes demographic information and open-ended questions about the factors affecting adherence to treatment in T2D patients. In order to improve the quality of the interview guide, the opinions of the initial participants are applied in the interview guide. Each interview begins by asking the main questions of the interview guide based on Pender’s HPM constructs. Then, exploratory questions are asked to elaborate on the issue. Probing questions are also used when necessary; probing questions provide an opportunity for the researcher to guide the interview and focus on the points of ambiguity. The interviews will be done in a place where participants feel comfortable and interview time will be about 40–60 min.

**Table 1:** Interview guide for the qualitative study

Opening questions	Probing questions
Please tell me about what is life like with diabetes?	What is the benefit of adhering to treatment as a diabetic person?
What factors influence your adherence to your treatment?	What are the barriers to adhering to treatment?
What do you know about the concept of adherence to treatment?	How capable you are of adhering to treatment?
	What do you feel about adherence to treatment?
	How effective are others to you about adherence to treatment?
	What effect does your work / living conditions have on your adherence to treatment?
	What do you do to commit to your treatment?

The rigor and credibility of the qualitative study will be evaluated according to Guba and Lincoln criteria [21]. The researcher will be engaged in the study process for a relatively long time. Findings and concepts will be shared with a number of participants to express their comments. Two out-of-study experts are asked to review the data. The researcher will use the maximum variation when selecting the study participants, and the study process will be explained in full detail.

#### Questionnaire design

The research team will categorize the results of the need assessment according to the constructs of HPM. Then, based on these categorized results, a treatment adherence questionnaire in accordance with HPM will design for T2D patients.

The questionnaire will be consisting of two parts: The first part is the demographic profile, which includes age, marriage, type of diabetes medication, age at the onset of diabetes, co-morbidities, and type of insurance covered. The second part will be based on constructs of HPM to measure adherence to medication, physical activity, and diet: perceived benefits of behavior, perceived barriers to behavior, perceived self-efficacy, activity-related affect, interpersonal and situational influences, immediate competing demands and preferences, commitment to plan of action, and health promotion behaviors.

The questionnaire, will be investigated and approved in terms of qualitative and quantitative content validity, face validity, construct validity through confirmatory factor analysis (CFA), and internal and external reliability.

#### Plans to promote participant retention and complete follow-up {18b}

Researchers will be in contact with participants through SMS and phone calls.

### Data management {19}

#### Qualitative stage

No name or surname in interviews (during the need assessment phase) will be recorded to protect the confidentiality of the participants’ information. After transcribing the interviews, the recorded information is deleted. The interview password-protected text is stored on a computer and is made only available to the first author. After gaining permission from the participants, all interviews are recorded using an audio-tape recorder and transcribed verbatim and then coded by the researchers through directed content analysis. The codes will be discussed, and finally, the codes will be categorized based on their similarities and concepts; therefore, them and sub them will make a construct according to HPM. Data management will be done by MAXQDA ver. 10.

#### Intervention stage

Training sessions during the intervention will be recorded using an audio-recorder that is encrypted and password-protected. Also, the files will transfer to a secure system. All identifiable data including any reference to the names or places will be removed from the data. Audio files will be saved in a separate password-protected folder and will be available only to the first author.

#### Confidentiality {27}

Participants’ personal identification information will be kept confidential and will be available to the clinic team and the first and second authors who will sign a non-disclosure agreement. Anonymous data will be available to other researchers of the study.

#### Plans for collection, laboratory evaluation, and storage of biological specimens for genetic or molecular analysis in this trial/future use {33}

There are no plans for collection, laboratory evaluation, and storage of biological specimens for genetic or molecular analysis in the current study and for future use in ancillary studies.

### Statistical methods

#### Statistical methods for primary and secondary outcomes {20a}

Data analysis will be performed using SPSS ver.26. To describe the quantitative variables, descriptive statistics will be conducted. In the intervention phase, independent *t*-test, paired *t*-test, and analysis of covariance are used to evaluate the effectiveness of the intervention on changing the behavior and HPM constructs.

#### Interim analyses {21b}

There are no interim analyses planned.

#### Methods for additional analyses (e.g., subgroup analyses) {20b}

A subgroup analyses will be performed for insulin to oral anti-diabetic drugs administrations.

#### Methods in analysis to handle protocol non-adherence and any statistical methods to handle missing data {20c}

In case of missing outcome data, the study analyses will be according to available data, relying on an assumption that data is missing at random. Reasons for missingness will be described by a chart, and frequency of miss patients for each stage will be described.

#### Plans to give access to the full protocol, participant level-data and statistical code {31c}

The protocol can be accessed via Iranian Registry of Clinical Trials (IRCT20211228053558N1: https://www.irct.ir/trial/61741). Reasonable access to other documents can be gained by contacting the corresponding author.

### Oversight and monitoring

#### Composition of the coordinating center and trial steering committee {5d}

The research team (consisting of three health education and promotion specialists, one doctor of internal medicine and one biostatistics expert) will be the supervision of all aspects of the study, including the completion of the study to ethical standards.

#### Composition of the data monitoring committee, its role and reporting structure {21a}

The Research Committee of Hormozgan University of Medical Sciences will ensure the safety of the study participants by monitoring the ethical conduct of the study to also ensure that the study is conducted according to the protocol and that the data are collected appropriately.

#### Adverse event reporting and harms {22}

This is not applicable as this is a low-risk intervention and no serious adverse events (SAE) are expected to occur in this trial on adherence.

#### Frequency and plans for auditing trial conduct {23}

A supervisor will review the existence and completeness of study records, such as informed consent forms, inclusion and exclusion criteria, and data collection and storage methods.

#### Plans for communicating important protocol amendments to relevant parties (e.g., trial participants, ethical committees) {25}

If necessary, any changes in the protocol modifications will be made with the consent of the ethic Committee of Hormozgan University of Medical Sciences and all authors and then, if finalized, will communicate to participants.

#### Dissemination plans {31a}

The findings will be disseminated to participants, Hormozgan University of Medical Sciences, health leads, health program policymakers, commissioners, and in peer-reviewed journals and academic conferences.

## Discussion

Behavior change is a complex activity that is not easy to accomplish, and even if it is accomplished, it is difficult to maintain new behavior [22] especially in T2D patients where treatment is related to their lifestyle. The present study will use HPM as a theory-based approach. Previous studies have shown the effectiveness of this model in treatment adherence among T2D patients. A quasi-experimental study is performed on T2D patients in Iran to use SMS based on HPM on the physical activity of diabetic patients. The results of the intervention group showed positive changes in perceived self-efficacy, family support, and perceived barriers [13]. The results of another study in Thailand showed the intervention group attained higher mean scores in terms of diabetes knowledge, perceived benefits, self-efficacy, activity-related affect, interpersonal and situational influences, commitment to plan of action, and health promotion behaviors as compared to the control group after the intervention [23]. Similar studies have demonstrated improvements in the physical activity [24] and nutrition [25] of non-patients based on HPM.

The importance of this study is that factors affecting treatment adherence in T2D patients are identified in different dimensions, so, we can design an effective relevant intervention through needs assessment and using the most appropriate methods to improve adherence to treatment in T2D patients in Bandar Abbas based on HPM. The results of this project can help policymakers and planners for macro-diabetic planning*.*

## Trial status

The current protocol is version number 1 of 17 March 2022. Expected recruitment for intervention start at November 2022. The study is anticipated to continue until March 2023.

## Supplementary Information

**Additional file 1.**

## Data Availability

Any data required to support the protocol can be supplied on request.

## Abbreviations

T2D: Type 2 diabetes mellitus
HPM: Pender’s health promotion model
HbA1C: Hemoglobin A1c test
CFA: Confirmatory factor analysis
SMS: Short message service
