# Supplementary material for: Application of Pender’s health promotion model for type 2 diabetes treatment adherence: protocol for a mixed methods study in southern Iran
Source: Trials. 2022 Dec 28;23:1056. doi: 10.1186/s13063-022-07027-9 (PMC9795658; doi:10.1186/s13063-022-07027-9)
Supplement: Supplementary file 1 — Additional file 1. [file 13063_2022_7027_MOESM1_ESM.docx]

|  | **Enrolment** | **Baseline** |  |  |  |  |  |  |  |  |  |  |  |
| --- | --- | --- | --- | --- | --- | --- | --- | --- | --- | --- | --- | --- | --- |
| **TIMEPOINT** | −*t*_1_ | 0 | S_1*_ | S_2_ | S_3_ | S_4_ | S_5_ | S_6_ | S_7_ | S_8_ | S_9_ | S _10_ | 3M** |
| **Need assessment** |  | X |  |  |  |  |  |  |  |  |  |  |  |
| **Instrument design** |  | X |  |  |  |  |  |  |  |  |  |  |  |
| **Eligibility screening** | X |  |  |  |  |  |  |  |  |  |  |  |  |
| **Informed consent** | X |  |  |  |  |  |  |  |  |  |  |  |  |
| **Randomization** |  | X |  |  |  |  |  |  |  |  |  |  |  |
| **intervention: Sessions** |  |  | X | X | X | X | X | X | X | X | X | X |  |
| **Assessment:** |  |  |  |  |  |  |  |  |  |  |  |  |  |
| **Questionnaire** |  | X |  |  |  |  |  |  |  |  |  | X | X |
| **HbA1c test** |  | X |  |  |  |  |  |  |  |  |  |  | X |
| **Attendance at clinic appointment** |  | X |  |  |  |  |  |  |  |  |  |  | X |
| **Normal range Fasting Blood Sugar days** |  | X |  |  |  |  |  |  |  |  |  |  | X |
| **weight** |  | X |  |  |  |  |  |  |  |  |  |  | X |
| **Healthy diets days** |  | X |  |  |  |  |  |  |  |  |  |  | X |
| **Physically active days** |  | X |  |  |  |  |  |  |  |  |  |  | X |

Legend: S* Session once a week, M** Month.
